# RETRACTED ARTICLE: RINT1 is a new suppression target to reduce colon cancer cell growth, migration and invasion through regulating ZW10/NAG-1 expression

**DOI:** 10.1007/s11010-020-03858-9

**Published:** 2020-08-04

**Authors:** Jinheng Xu, Meng Zhao, Shunxian Huang, Qian Wu, Minghe Bai, Xueli Zhao, Jixian Wang, Yueming Hu, Junwei Feng, Zhiyong Zhang

**Affiliations:** 1 Department of Pathology, Tangshan Gongren Hospital, Lubei District, No. 27 Wenhua Road, Tangshan, 063000 Hebei China; 2 Department of Biological Science, College of Life and Environmental Sciences, Minzu University of China, Beijing, 100081 China; 3 Department of Oncological Surgery, Tangshan Gongren Hospital, Lubei District, No. 27 Wenhua Road, Tangshan, 063000 Hebei China

The Editor in Chief retracted this article because of significant concerns regarding figures 4 and 8 presented in this work, which question the integrity of the data. All authors agree to this retraction. The online version of this article contains the full text of the retracted article as Supplementary Information.

## Supplementary Information

Below is the link to the electronic supplementary material.

Supplementary file1 (PDF 0 kb)

