# Supplementary material for: RETRACTED ARTICLE: RINT1 is a new suppression target to reduce colon cancer cell growth, migration and invasion through regulating ZW10/NAG-1 expression
Source: Mol Cell Biochem. 2020 Aug 4;477(11):2683. doi: 10.1007/s11010-020-03858-9 (PMC9618504; doi:10.1007/s11010-020-03858-9)
Supplement: Supplementary file 1 — Supplementary file1 (PDF 0 kb) [file 11010_2020_3858_MOESM1_ESM.pdf]

Molecular and Cellular Biochemistry
https://doi.org/10.1007/s11010-020-03858-9

RETRACTED ARTICLE

RINT1 is a new suppression target to reduce colon cancer cell growth, migration and invasion through regulating ZW10/NAG-1 expression

Jinheng Xu[1] · Meng Zhao[1] · Shunxian Huang[2] · Qian Wu[1] · Minghe Bai[1] · Xueli Zhao[1] · Jixian Wang[1] · Yue[illegible]ng Hu[1] · Junwei Feng[3] · Zhiyong Zhang[1]

Received: 13 April 2020 / Accepted: 24 July 2020
© The Author(s) 2020

Abstract

This study explored the function and mechanism of RAD50 interactor 1 (RINT1), [illegible]te white 10 (ZW10) or nonsteroidal anti-inflammatory drug-activated gene-1 (NAG-1) in cell growth of colon cancer. The m[illegible]A expression of RINT1 was suppressed in patients with colon cancer. In addition, disease free survival (DF[illegible]) in [illegible]olon cancer patients with high expression of RINT1, ZW10 and NAG-1 were promoted compared with colon cancer p[illegible]ith low expression of RINT1, ZW10 and NAG-1. Over-expression of RINT1, ZW10 or NAG-1 reduced colon cance[illegible]l growth, migration and invasion in vitro model. In contrast, down-regulation of RINT1, ZW10 or NAG-1 prom[illegible]olon cancer cell growth, migration and invasion in vitro model. Over-expression of RINT1 promoted while down-regulation of RINT1 suppressed ZW10 and NAG-1 expression. Following knockdown of ZW10 and NAG-1 protein expressions, RI[illegible]T1 induced changes of colon cancer cell growth, migration and invasion were inhibited. Taken together, it i[illegible]ted that RINT1 is a new suppression target to reduce cell growth, migration and invasion of colon cancer by targe[illegible] ZW[illegible]/NAG-1.

Keywords RINT1 · ZW10 · NAG-1 · Cell growth · [illegible]ol[illegible]n cancer

Introduction

Colorectal cancer (CRC) is one of t[illegible] common malignancies [1]. The incidence rate ranks t[illegible]third [illegible]n males and the second in females worldw[illegible] and the morbidity and mortality are gradually increasing [illegible]sing serious threat to human health [1]. Th[illegible]cidence is relatively higher in populations over 50 y[illegible]rs [illegible] and relatively lower in those under 50 years old [illegible]2]. A[illegible]rding to reports from developed countries such a[illegible]e United States and Australia, due to the popularity of CRC screening in recent years, the incidence of CRC in the United States has declined [3]. The incidence of CRC in males and females has decreased by 2.8% and 2.2% annually on average, respectively. However, the incidence of CRC in young adults is increasing. The incidence of CRC among young males and females in the 20–49 age group is increased by 1.5% and 1.6%, respectively per year in the United States [3].

Zeste white 10 (ZW10) is a highly evolutionarily-conserved protein which was originally found in Drosophila cells [4]. Subsequent studies have demonstrated the wide distribution of ZW10 in multicellular eukaryotes [4, 5]. DNA damage repair is a protective mechanism of cells and also an important pathway of tumor apoptosis [6]. Neuroblastoma-amplified gene-1 (NAG-1), a member of the TGF-β superfamily, is also known as macrophage inhibitory cytokine-1, placental transforming growth factor-β, prostate-derived factor, placental bone morphogenetic protein, or growth differentiation factor-15 (GDF-15) [7, 8]. NAG-1 is involved in the carcinogenesis and tumor progression [9]. Changes in NAG-1 gene expression can be detected in multiple types of tumors [10]. Although NAG-1 has been reported as a component of anti-tumor complex to

Jinheng X[illegible] and Zhiyong Zhang are sharing first authorship.

✉ Zhiyong [illegible]ng
zh[illegible]ngzhan[illegible]23@163.com

1 [illegible]artment of Pathology, Tangshan Gongren Hospital, Lu[illegible] District, No. 27 Wenhua Road, Tangshan 063000, Hebei, China

2 Department of Biological Science, College of Life and Environmental Sciences, Minzu University of China, Beijing 100081, China

3 Department of Oncological Surgery, Tangshan Gongren Hospital, Lubei District, No. 27 Wenhua Road, Tangshan 063000, Hebei, China

participate in tumor apoptosis, the mechanism involved is extremely complicated and might change according to tumor microenvironment [9, 11].

DNA damage repair process is an important research field in tumor biology. Multiple classical genes involved in DNA damage response, such as P53 and breast cancer susceptibility gene-1 (BRCA1), play roles as tumor suppressor genes [12, 13]. RINT1 is critically involved in the repair of double-strand breaks, which also participates in the proliferation and apoptosis of various types of tumors [12]. This study explored the function and mechanism of RINT1, ZW10 or NAG-1 in cell growth, migration and invasion of colon cancer.

Materials and methods

Clinical samples

From March 2013 to September 2014, colon cancer tissue and normal colon tissue samples were obtained from 42 patients who were diagnosed with colon cancer at Tangshan Gongren Hospital, China. Tissues were immersed in liquid nitrogen and were stored at − 80 °C until use. Approval for this study was provided by the Ethics Committee of Tangshan Gongren Hospital, China. Informed consent was obtained from patients.

RNA extraction and quantitative real-time polymerase chain reaction (qRT-PCR)

Total RNA was extracted from colon cancer tissue samples using TRIzol reagent (Thermo Fisher Scientific, Inc., Waltham, MA, USA). cDNA was synthesized using High-Capacity cDNA Reverse Transcription kit (Applied Biosystems, Foster City, CA, USA). qRT-PCR was performed using an ABI 7900 PCR Thermal Cycler with a SYBR Green kit (Bio-Rad Laboratories, Inc., Hercules, CA, USA). The mRNA expression levels of genes were measured using the $2^{-\Delta\Delta C_t}$ method.

Cell Culture and transfection

[illegible] cell line was obtained from the Cell Bank (Shanghai Genechem Co., Ltd., Shanghai, China). Cells were cultured in Dulbecco's modified eagle's medium (DMEM; Gibco, Grand Island, NY, USA) supplemented with 10% fetal bovine serum (FBS; Gibco, Grand Island, NY, USA) in an incubator at 37 °C with 5% CO_2. Following 24 of culture, cells were transfected with RINT1, ZW10 and NAG-1 mimics, and si-RINT1, si-ZW10, si-NAG-1 plasmids using Lipofectamine^{2000} (Invitrogen, Carlsbad, CA, USA).

Cell viability and cytotoxicity

Cell viability was analyzed using methylthiazolyldiphenyltetrazolium bromide (MTT) assays (Beyotime, Nantong, China) at 0, 24, 48 and 72 h after transfection. A total of 20 μL of MTT solution was added to each well and incubated for 4 h at 37 °C. Supernatants were removed and formazan crystals were dissolved in 150 μL dimethylsulfoxide (DMSO). Absorbance was measured using a Multiskan Spectrum Microplate Spectrophotometer (Thermo Scientific™, Waltham, MA, USA) at a wavelength of 492 nm.

Cytotoxicity for analyzing Lactate dehydrogenase (LDH) activity was measured using LDH activity kits (Beyotime, Nantong, China). Absorbance was measured using a Multiskan Spectrum Microplate Spectrophotometer (Thermo Scientific™, Waltham, MA, USA) at a wavelength of 450 nm.

Scratch test

A total of 100 μL of transfection cells (1×10^6/mL) were resuspended in serum-free medium and seeded onto each well of 6 well plates. Then, 200 μL tips were used to scratch and cells were observed using microscope at 24 h after scratch.

Western blot analysis

Total proteins of cells were collected using Radio-immunoprecipitation assay (RIPA) lysis buffer and protease inhibitor cocktail (PMCF; 1:100, Beyotime, Nantong, China), and the concentration of these protein was determined by using BCA Kit (Beyotime, Nantong, China). Then, 50 μg protein samples were loaded to 10% sodium dodecyl sulfate–polyacrylamide gel electrophoresis (SDS-PGE), and then transferred onto polyvinyl difluoride (PVDF; Thermo Scientific™, Waltham, MA, USA) membranes. Membranes were incubated with RINT1, ZW10, NAG-1, TGF-β and GAPDH antibodies at 4 °C overnight after blocking with 5% non-fat milk in tris-buffered saline with 0.1% tween 20 (TBST). The membranes were washed with TBST for three times and then incubated with anti-rabbit secondary antibody (1:5000) for 2 h at room temperature. All antibodies were purchased from Cell Signaling Technology (Beverly, MA, USA). Immunoreactive bands were visualized using the ECL kit (Thermo Scientific™, Waltham, MA, USA) and integrated density of the bands was quantified by Quantity One software 3.0 (Bio-Rad, CA, USA).

RETRACTED ARTICLE

RETRACTED ARTICLE

Statistical analysis

Results are expressed as means ± standard deviation (SD). Statistical analysis was performed with the Student unpaired t test or One-way analysis of variance (ANOVA) and Tukey's post test in Statistical Product and Service Solutions (SPSS) version 19.0 (SPSS Inc., Chicago, IL, USA). p values < 0.05 were considered to indicate statistical significance.

Results

RINT1, ZW10 and NAG-1 expression observed in colon cancer patients

The mRNA expression of RINT1, ZW10 and NAG-1 in normal tissues and cancer tissues of colon cancer patients was determined by qRT-PCR. It was found that mRNA expression of RINT1 (Fig. 1a, b), ZW10 (Fig. 1d, e) and and NAG-1 (Fig. 1g, h) was reduced in colon cancer tissues of patients, compared with normal tissues of patients ($p < 0.01$). mRNA expression of RINT1 (Fig. 1c), ZW10 (Fig. 1f) and NAG-1 (Fig. 1i) in tissues of patients with stage IV colon cancer was lower than that of patients with stage III colon cancer ($p < 0.01$). In addition, tissue mRNA expression of RINT1 (Fig. 1c), ZW10 (Fig. 1f) and NA[illegible] (Fig. 1i) in patients with stage III colon cancer was l[illegible]er [illegible]han that of patients with stage I-II colon cancer ($p < 0.0$[illegible]

Effects of RINT1, ZW10 and NAG-[illegible]n the survival rate of colon cancer patien[illegible]s

The effects of RINT1, Z[illegible]10 a[illegible]NAG-1 on the survival rate of colon cancer patien[illegible]s w[illegible] investigated. It was suggested that overall surviva[illegible]OS) in c[illegible]n cancer patients with high expression of RI[illegible]T1 ([illegible]g. 2a), ZW10 (Fig. 2c) and NAG-1 (Fig. 2e) was [illegible]milar to patients with low expression of RINT1, Z[illegible]10 a[illegible]NAG-1. However, DFS in colon cancer patients with [illegible]h expression of RINT1, ZW10 and NAG-1

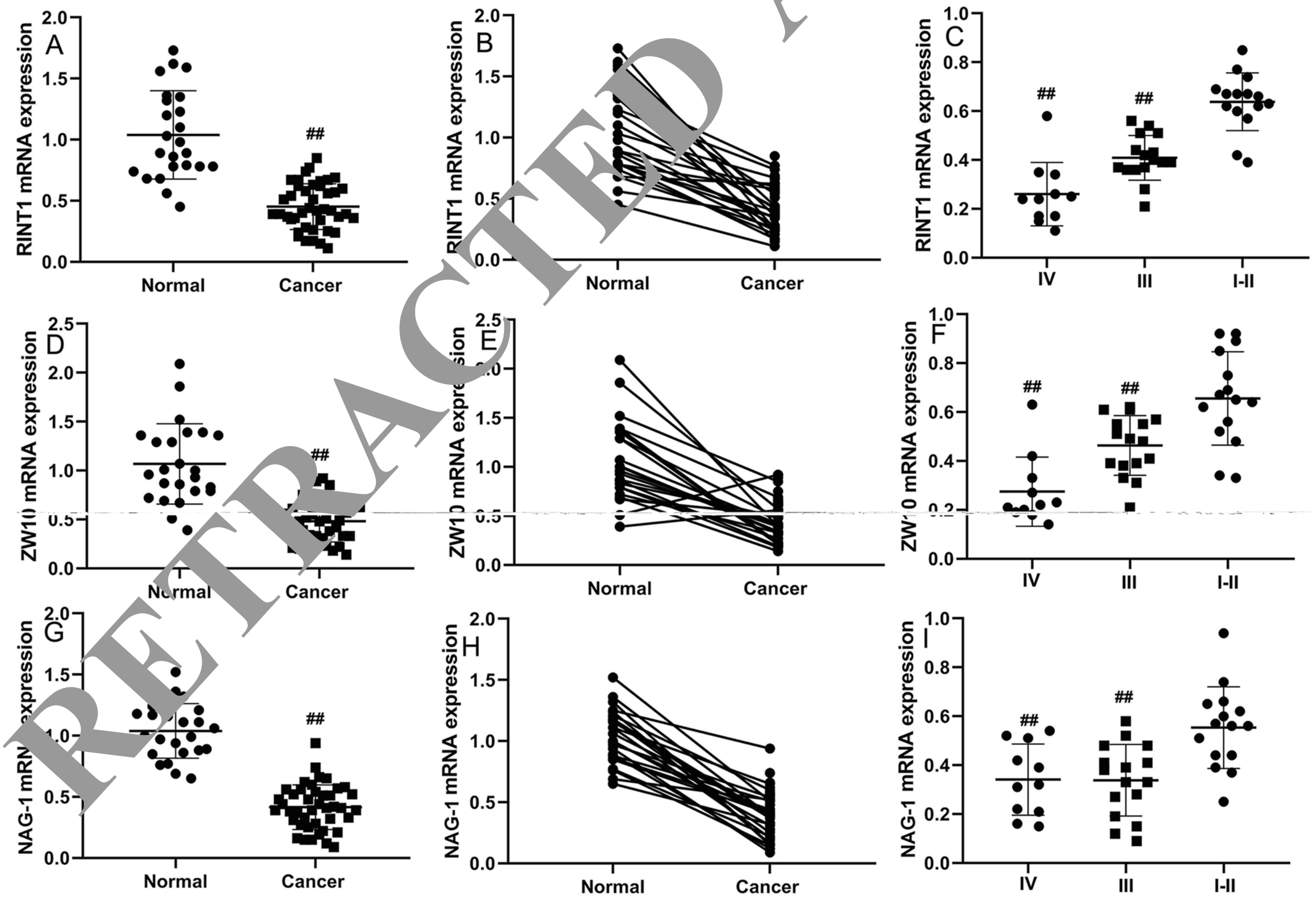

Fig. 1 RINT1, ZW10 and NAG-1 expression was observed in colon cancer patients. RINT1 mRNA expression (**a**, **b** and **c**) in colon cancer patients; ZW10 mRNA expression (**d**, **e** and **f**) in colon cancer patients; NAG-1 mRNA expression (**g**, **h** and **i**) in colon cancer patients. Normal, normal tissues; Cancer, colon cancer tissues. $n = 42$ patients

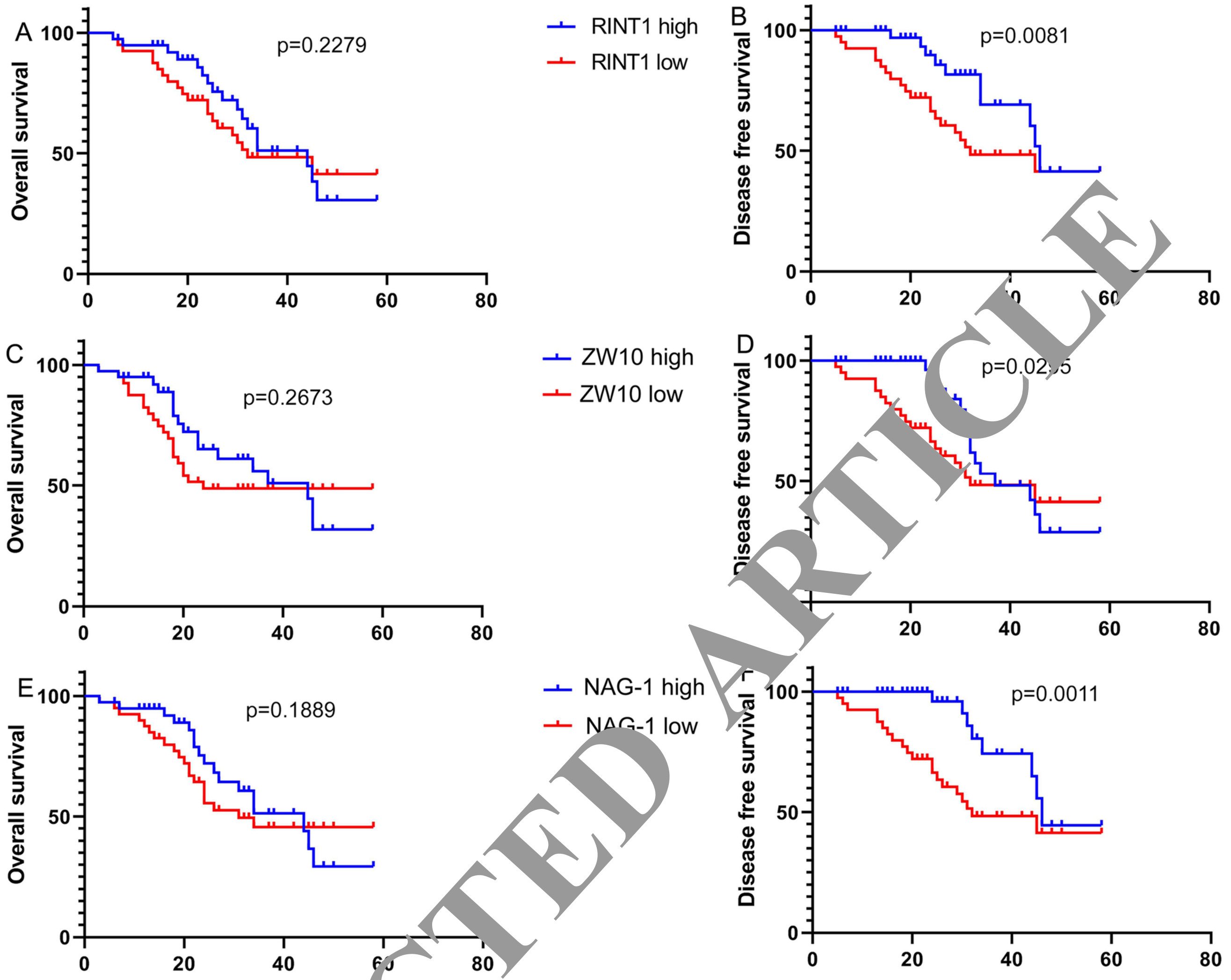

Fig. 2 Survival rate of colon cancer patients with high or low RINT1, ZW10 or NAG-1 expression. OS (**a**) and DFS (**b**) of colon cancer patients with higher or lower RINT1 expression. OS (**c**) and DFS (**d**) of colon cancer patients with higher or lower ZW10 expression. OS (**e**) and DFS (**f**) of colon cancer patients with high or low NAG-1 expression. *RINT1 high* RINT1 higher expression, *RINT1 low* RINT1 lower expression, *ZW10 high* ZW10 higher expression, *ZW10 low* ZW10 lower expression, *NAG-1 high* NAG-1 higher expression, *NAG-1 low* NAG-1 lower expression. $^{\#\#}p<0.01$ compared with negative group. $n=42$ patients. *OS* overall survival, *DFS* disease free survival

RETRACTED ARTICLE

were higher than colon cancer patients with low expression of RINT1 (Fig. 2b), ZW10 (Fig. 2d) and NAG-1 (Fig. 2f).

RINT1 regulated growth, migration and invasion of colon cancer cells

To confirm the effects of RINT1 on colon cancer growth, migration and invasion, RINT1-mimic plasmids were transfected into HCT 116 cells. The expression of RINT1 was increased in cultured cancer cells, compared with the negative group ($p<0.01$; Fig. 3a). It was found that, compared with the negative control, over-expression of RINT1 reduced cell growth ($p<0.01$; Fig. 3b), increased cytotoxicity ($p<0.01$; Fig. 3c), suppressed migration ($p<0.01$; Fig. 3d, e) and invasion rate ($p<0.01$; Fig. 3f, g) and increased caspase-3/9 activity levels ($p<0.01$; Fig. 3h, i) in vitro model of colon cancer.

To further prove the results of RINT1, si-RINT1 was used to treat with HCT 116 cells. RINT1 expression was heavily reduced in si-RINT1 transfected colon cancer cells, in comparison with the negative group ($p<0.01$; Fig. 3j). The down-regulation of RINT1 promoted cell growth ($p<0.01$; Fig. 3k), accelerated migration ($p<0.01$; Fig. 3l, m) and invasion rate ($p<0.01$; Fig. 3n, o), reduced cytotoxicity ($p<0.01$; Fig. 3p), and decreased caspase-3/9 activity levels ($p<0.01$; Fig. 3q, r) in vitro model of colon cancer, compared with the negative group.

RETRACTED ARTICLE

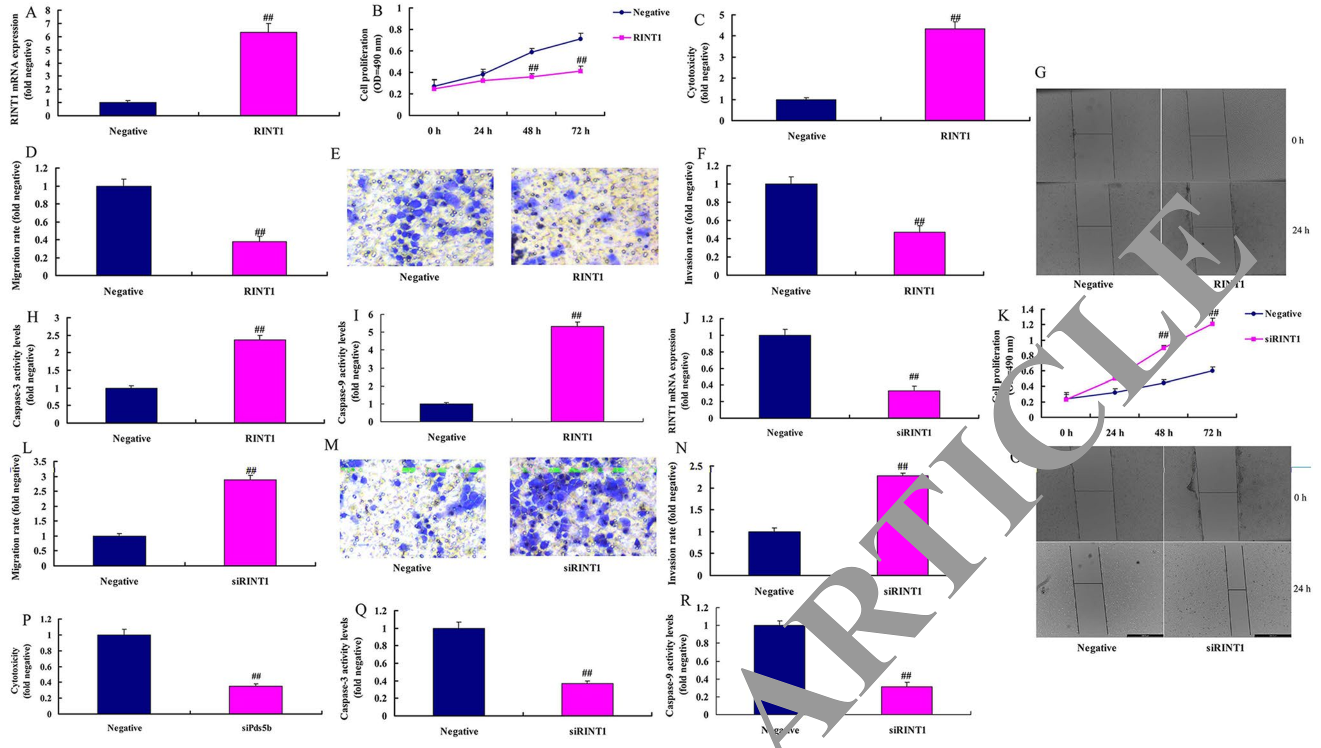

Fig. 3 RINT1 regulated cell growth, migration and invasion of colon cancer. Changes of RINT1 mRNA expression (**a**), cell viability (**b**), cytotoxicity (**c**), migration rate (**d** and **e**), invasion rate (**f** and **g**), caspase-3/9 activity levels (**h** and **i**) in vitro model induced by over-expression of RINT1; changes of RINT1 mRNA expression (**j**), cell viabiliy (**k**), cytotoxicity (**l**), migration rate (**m** and **n**), invasion rate (**o** and **p**), caspase-3/9 activity levels (**q** and **r**) in vitro model induced by down-regulation of RINT1. *Negative* negative mimics group, *RINT1* over-expression of RINT1 group, *si-RINT1* down-regulation of RINT1 group. $^{\#\#}p<0.01$ compared with negative group

ZW10 regulated growth, migration and invasion of colon cancer cells

This study further confirmed the function of ZW10 in cell growth, migration and invasion of colon cancer. As shown in Fig. 4a, ZW10 mimic plasmid significantly promoted ZW10 expression in cultured colon cancer cells ($p<0.01$). Furthermore, compared to the negative control, over-expression of ZW10 reduced cell growth ($p<0.01$; Fig. 4b), increased cytotoxicity ($p<0.01$; Fig. 4c), reduced migration ($p<0.01$; Fig. 4d, e) and invasion rate ($p<0.01$; Fig. 4f, g) and increased caspase-3/9 activity levels ($p<0.01$; Fig. 4h, i) in colon cancer cells. In contrast, si-ZW10 suppressed ZW10 expression in vitro model of colon cancer, compared with the negative group ($p<0.01$; Fig. 4j). ZW10 down-regulation promoted cell growth ($p<0.01$; Fig. 4k), accelerated migration ($p<0.01$; Fig. 4l, m) and invasion rate ($p<0.01$; Fig. 4n, o), reduced cytotoxicity ($p<0.01$; Fig. 4p), and decreased caspase-3/9 activity levels ($p<0.01$; Fig. 4q, r) in colon cancer cells, compared with the negative group.

NAG-1 regulated growth, migration and invasion of colon cancer cells

This study confirmed that the anti-effects of NAG-1 in cell growth, migration and invasion of colon cancer. Compared to the negative control, NAG-1 mimic plasmid induced higher NAG-1 expression in vitro cultured colon cancer cells ($p<0.01$; Fig. 5a). Over-expression of NAG-1 reduced cell growth ($p<0.01$; Fig. 5b), increased cytotoxicity ($p<0.01$; Fig. 5c), reduced migration ($p<0.01$; Fig. 5d, e) and invasion ($p<0.01$; Fig. 5f, g) rate and increased caspase-3/9 activity levels ($p<0.01$; Fig. 5h, i) in vitro model of colon cancer, compared with negative group. Moreover, si-NAG-1 decreased NAG-1 expression significantly in vitro model of colon cancer, indicating the good efficiency of si-NAG-1 ($p<0.01$; Fig. 5j). NAG-1 down-regulation promoted cell growth ($p<0.01$; Fig. 5k), accelerated migration ($p<0.01$; Fig. 5l, m) and invasion ($p<0.01$; Fig. 5n, o), reduced cytotoxicity ($p<0.01$; Fig. 5p), and decreased caspase-3/9 activity levels ($p<0.01$; Fig. 5q, r) in colon cancer cells, compared with the negative group.

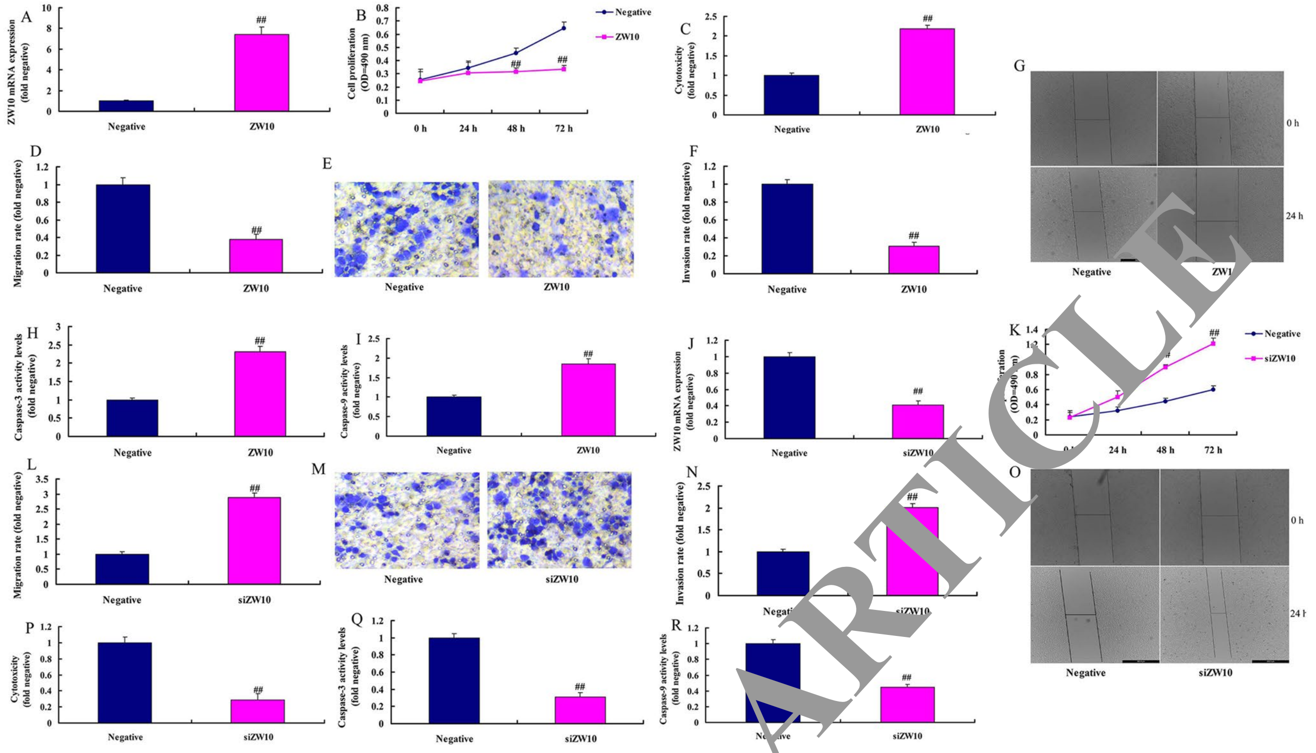

Fig. 4 ZW10 regulated cell growth, migration and invasion of colon cancer. Changes of ZW10 mRNA expression (**a**), cell viability ([illegible]), cytotoxicity (**c**), migration rate (**d** and **e**), invasion rate (**f** [illegible] **g**), caspase-3/9 activity levels (**h** and **i**) in vitro model induced by over-expression of ZW10; changes of ZW10 mRNA expressi[illegible] (**j**), cell [illegible]liferation (**k**), cytotoxicity (**l**), migration rate (**m** and **n**), invasion ra[illegible] (**o** and **p**), caspase-3/9 activity levels (**q** and **r**) in vitro model in[illegible]uced by down-regulation of ZW10. *Negative* negative mimics [illegible]roup, *ZW10* over-expression of NAG-1 group, *si-NAG-1* down-regulation of NAG-1 group. $^{\#\#}p<0.01$ compared with negative group

RINT1 regulated ZW10/ NAG-1 expr[illegible]sion i[illegible]lon cancer cells

To determine the mechanism of RINT1 [illegible]olon cancer, we used gene chip to analyze the ex[illegible]ion gene regulated by RINT1. As showed in Fig. 6a, RI[illegible]T1 might regulate ZW10/ NAG-1 to reduce reac[illegible]ive [illegible]xygen species (ROS) production. Over-expression [illegible] R[illegible] promoted ZW10 (Fig. 6b, d) and NAG-1 (Fi[illegible]. 6b, c) [illegible]otein expressions in colon cancer cell line, c[illegible]mp[illegible]ed with the negative group ($p<0.01$). Conversely [illegible]ompare[illegible] the negative control, down-regulation of R[illegible]NT1 obviously suppressed ZW10 (Fig. 6e, f) and NAG-1 (F[illegible]6e, g) protein expressions in colon cancer cell line [illegible]0.01).

The [illegible]hibition of ZW10/ NAG-1 suppressed the ef[illegible]ects of RINT1 on growth of colon cancer cells

To further analyze the role of ZW10 in the effects of RINT1 on growth of colon cancer cells, the expression of ZW10 was inhibited using si-ZW10. As shown in Fig. 7a–c, compared with the RINT1 over-expression group, si-ZW10 suppressed ZW10 and NAG-1 protein expressions induced by over-expression of RINT1 in cultured colon cancer cells ($p<0.01$). Furthermore, RINT1 over-expression induced changes of cell viability (Fig. 7d), migration (Fig. 7e, f), invasion (Fig. 7g, h), cytotoxicity (Fig. 7i), and caspase-3/9 activity (Fig. 7g, k) of colon cancer cells were significantly inhibited following the inhibition of ZW10 ($p<0.01$).

It was indicated that RINT1 changed growth of colon cancer cells by the inhibition of ZW10.

Next, the role of NAG-1 in the effects of RINT1 on growth of colon cancer cells was further determined by si-NAG-1. It was shown that si-NAG-1 suppressed NAG-1 protein expressions induced by over-expression of RINT1in colon cancer cells (Fig. 8a, b). The inhibition of NAG-1 promoted cell growth (Fig. 8c), migration (Fig. 8d, e) and invasion (Fig. 8g, h), and inhibited cytotoxicity (Fig. 8f) and caspase-3/9 activity (Fig. 8i, j) in vitro model of colon cancer induced by over-expression of RINT1 ($p<0.01$).

RETRACTED ARTICLE

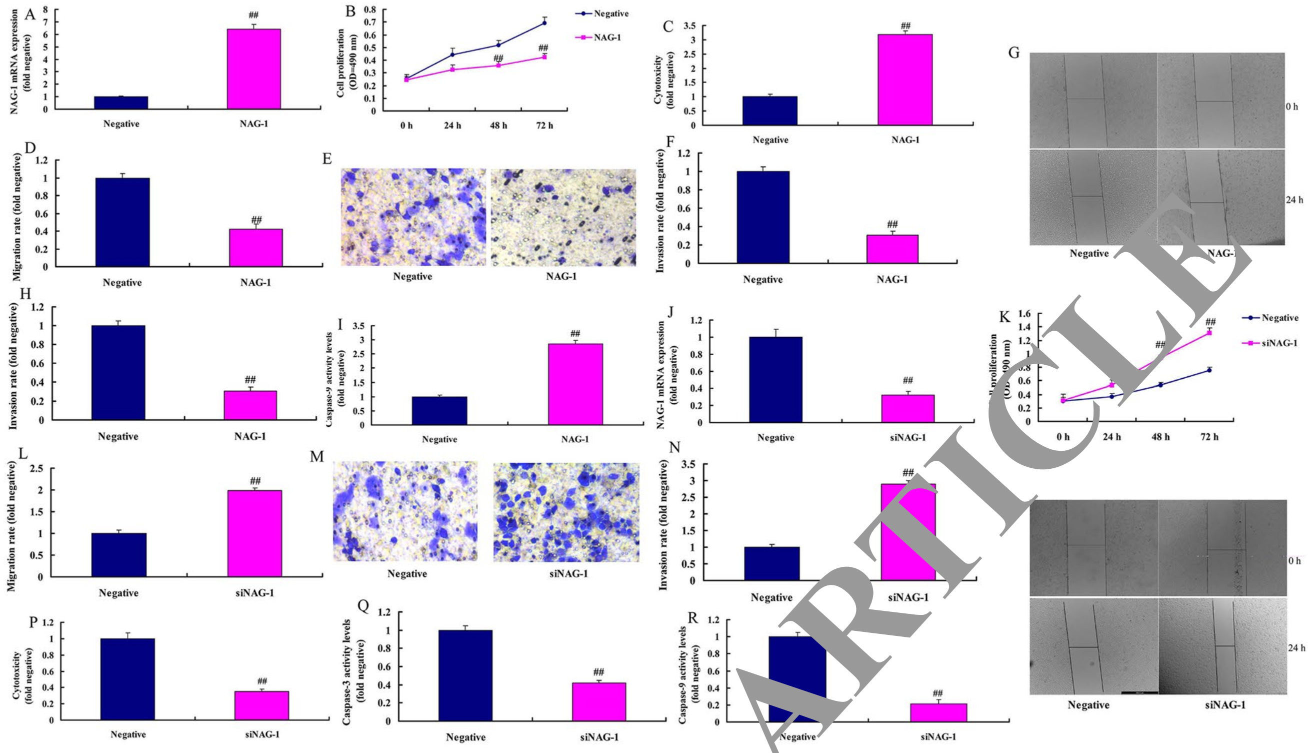

Fig. 5 NAG-1 regulated cell growth, migration and invasion of colon cancer. NAG-1 mRNA expression (**a**), cell viability (**b**), cytotoxicity (**c**), migration rate (**d** and **e**), invasion rate (**f** and **g**), caspase-3/9 activity levels (**h** and **i**) in vitro model were regulated by over-expression of NAG-1; NAG-1 mRNA expression (**j**), cell viability (**k**), cytotoxicity (**l**), migration rate (**m** and **n**), invasion rate (**o** and **p**), caspase-3/9 activity levels (**q** and **r**) in vitro model were regulated by down-regulation of NAG-1. *Negative* negative mimics group, *NAG-1* over-expression of NAG-1 group < *si-NAG-1* down-regulation of NAG-1 group. $^{\#\#}p < 0.01$ compared with negative group

RETRACTED ARTICLE

Discussion

Compared with CRC in old population, the incidence of CRC in young population is relatively low. In general, CRC is not common in population under 40 years old [14, 15]. The risk of CRC under 40 years old is 1:1 200, while it rises to 1:25 in population over 70 years old [15]. In recent years, due to the popularity of CRC screening in the elderly, the incidence of CRC in the old population in the United States, Europe and other developed Western countries has gradually decreased [[illegible]]. However, the incidence and mortality of CRC in young people has been increasing, and CRC has become the third leading cause of death following leukemia and neurological diseases among population within 2[illegible] years old [14]. This study proved that down-regulation of RINT1, ZW10 and NAG-1 expression was observed in colon cancer patients. RINT1 reduced colon cancer cell growth, migration and invasion by targeting ZW10 or NAG-1.

RINT1 was first identified as a RAD50-interacting protein of 792 amino acids. An N-terminally truncated RINT1 protein over-expression results in a defect in the radiation-induced G2/M checkpoint, indicating that RINT1 plays an important role in cell cycle progression [17]. Lin et al. reported that RINT1 serves as a tumor suppressor and maintains Golgi dynamics for cell survival [18]. This study also suggested that RINT1 expression is decreased in patients with colon cancer, and RINT1 reduces colon cancer cell growth, migration and invasion.

NAG-1 is associated with multiple diseases including cancer. The expression of NAG-1 is lower in glioblastoma cell lines than that in benign glioma cell lines and normal human astrocytes [19]. Existing in vitro studies have shown suggest that NAG-1 expression inhibits tumor cell growth [19–21]. A study provided the evidence that over-expression

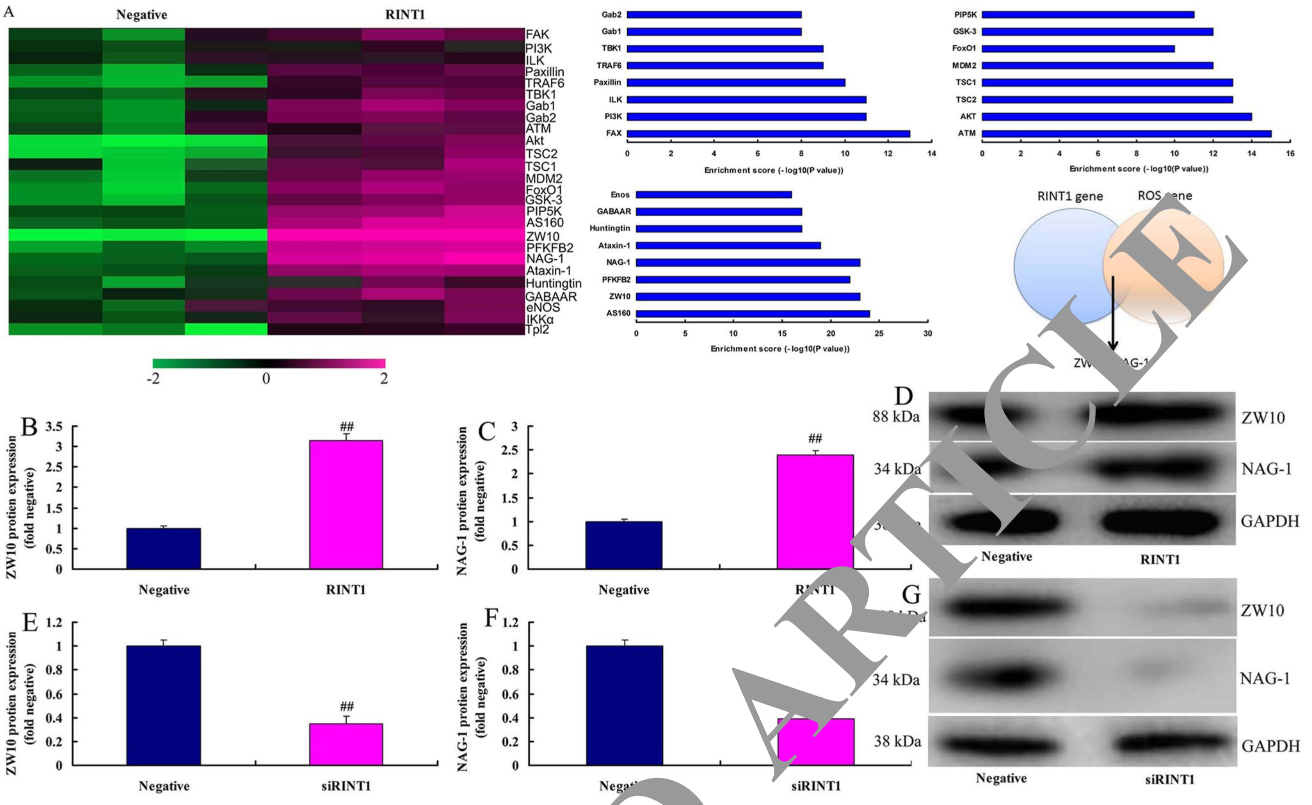

Fig. 6 RINT1 regulated ZW10/ NAG-1 expression in colon cancer cells. Gene chip (**a**) and analyze results (**b**); changes of ZW10 and NAG-1 protein expression by over-expression of RINT1 (c, d and e); changes of ZW10 and NAG-1 protein expression by down-regulation of RINT1 (f, g and h). *Negative* negative mimics group, *ZW10* over-expression of NAG-1 group, *si-NAG-1* down-regulation of NAG-1 group. $^{\#\#}p < 0.01$ compared with negative group

RETRACTED ARTICLE

of NAG-1 can inhibit the proliferation of breast cancer cells, epithelial tumor cells and prostate cancer cells [22]. In addition, the tumor formation ability of human colon cancer and glioma cells with NAG-1 overexpression is significantly decreased in nude mice [23]. Further studies have demonstrated that overexpression of NAG-1 can cause caspase-dependent apoptosis in prostate cancer cells [24]. Above results suggest that NAG-1 is closely associated with tumorigenesis. In this study, we also found that NAG-1 can suppress colon cancer cell viability, migration and invasion, and its protein expression can be regulated by RINT1.

ZW10 plays an extremely important role in both mitosis and meiosis [24]. ZW10 mutations exert similar effects on mitosis and meiosis, causing delayed chromosome and chromosomal unequal separation, ultimately regulating tumor proliferation and apoptosis [25]. We also identified

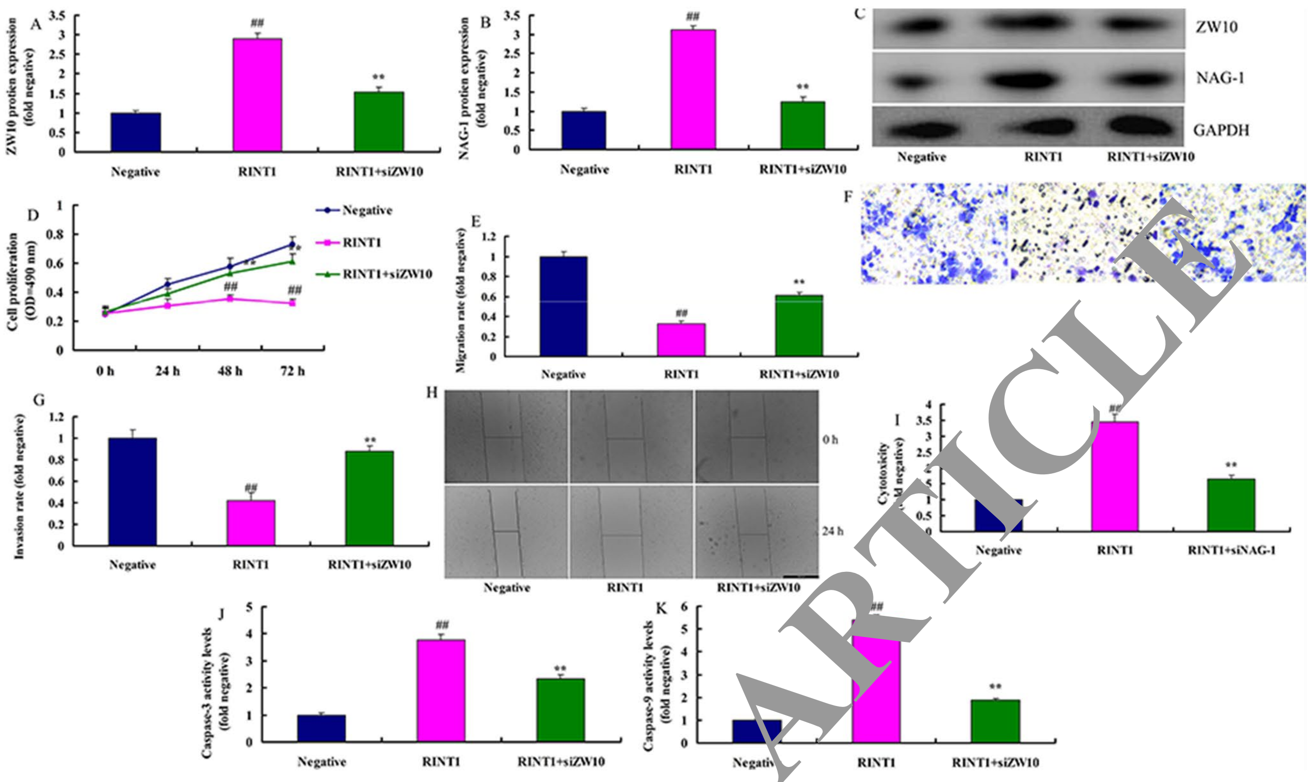

Fig. 7 The inhibition of ZW10 reduced the effects of RINT1 on [illegible]ll growth, migration and invasion of colon cancer. ZW10 and NAG-[illegible] protein expression (**a**, **b** and **c**), cell viability (**d**), migratio[illegible] rate (**e** and **f**), invasion rate (**g** and **h**), cytotoxicity (**i**), and caspas[illegible]/9 [illegible]tivity levels (**j** and **k**). Negative, negative mimics group; [illegible]IN[illegible] over-e[illegible]ression of RINT1 group; RINT1 + si-ZW10, over-expression of RI[illegible]T1 and down-regulation of ZW10 group. $^{\#\#}p<0.01$ compared [illegible]ith negative group, $^{**}p<0.01$ compared with over-expression of RINT1 group

the inhibition of ZW10 or NAG-1 red[illegible]ed the [illegible]ffects of RINT1 on cell growth of colon cancer[illegible] Arasaki et al. showed that RINT1 coordinates the localiz[illegible]ion and function of ZW10 at the ER and the Golgi appara[illegible]hroughout the cell cycle [26]. This investigatio[illegible]gests that RINT1 activation may be associated with ZW1[illegible]/N[illegible]AG-1 and subsequent improvement of colon [illegible]r.

Conclusions

In summary, our data indicate that RINT1, ZW10 and NAG-1 expression were down-regulated in colon cancer patients. Over-expression of RINT1, ZW10 or NAG-1 reduced cell viability, migration and invasion in vitro model. In addition, RINT1 induced ZW10 and NAG-1 protein expressions to control colon cancer cell growth, migration and invasion. RINT1,

RETRACTED ARTICLE

RETRACTED ARTICLE

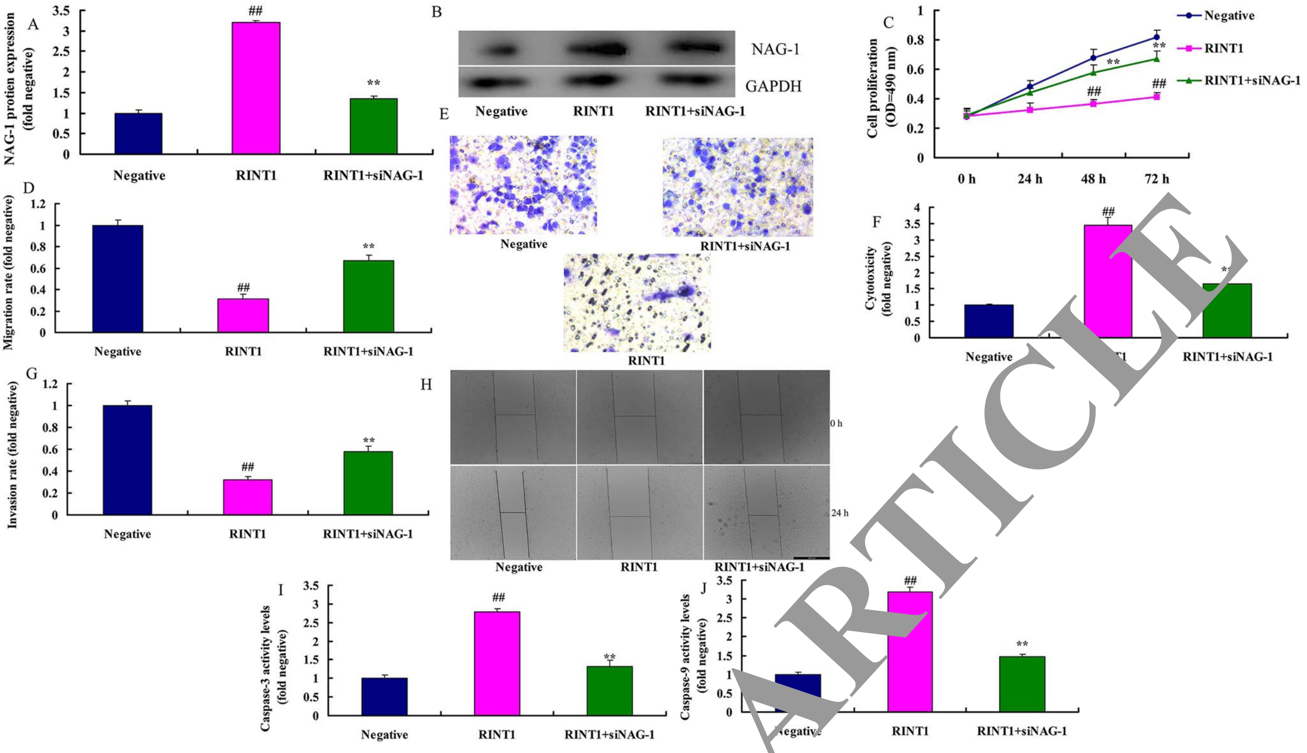

Fig. 8 The inhibition of NAG-1 reduced the effects of RINT1 o[illegible] cell growth, migration and invasion of colon cancer. NAG-1 pro[illegible] expression (**a** and **b**), cell viability (**c**), migration rate (**d** and **e**[illegible] cytotoxicity (**f**), invasion rate (**g** and **h**), caspase-3/9 activity lev[illegible] (**i** and [illegible] *Negative* negative mimics group, *RINT1* over-expression of RINT1 g[illegible]up, *RINT1 + si-NAG-1* over-expression of RINT1 and down-regul[illegible]tion of NAG-1 group. ##$p < 0.01$ compared with negative group, [illegible]*$p < 0.01$ compared with over-expression of RINT1 group

ZW10 and NAG-1 may help lead to the dev[illegible]ent of new drugs to cure colon cancer.

Funding This work was supported by the [illegible] K[illegible]y Research and Development Program: Joint Resea[illegible] on Accurate Diagnosis and Treatment of Colorectal Cancer (18[illegible]). Thanks to the Key Research and Development Program of [illegible]angshan City: Study on Intelligent Interpretation System of [illegible]mph N[illegible]de Metastasis of Colorectal Cancer Based on Deep L[illegible]

Compliance w[illegible] ethical standards

Conflict of i[illegible]terest The au[illegible]hors declare that there are no potential conflicts of i[illegible]est[illegible] dis[illegible]lose.

Ethi[illegible]pprova[illegible] [illegible]he study was approved by the Tangshan Gongren H[illegible]pita[illegible] China.

Inform[illegible]consent Informed consent was obtained from patients.

Open Access This article is licensed under a Creative Commons Attribution 4.0 International License, which permits use, sharing, adaptation, distribution and reproduction in any medium or format, as long as you give appropriate credit to the original author(s) and the source, provide a link to the Creative Commons licence, and indicate if changes were made. The images or other third party material in this article are included in the article's Creative Commons licence, unless indicated otherwise in a credit line to the material. If material is not included in the article's Creative Commons licence and your intended use is not permitted by statutory regulation or exceeds the permitted use, you will need to obtain permission directly from the copyright holder. To view a copy of this licence, visit http://creativecommons.org/licenses/by/4.0/.

References

1. Rutegard MK, Batsman M, Axelsson J, Brynolfsson P, Brannstrom F, Rutegard J, Ljuslinder I, Blomqvist L, Palmqvist R, Rutegard M, Riklund K (2019) PET/MRI and PET/CT hybrid imaging of rectal cancer—description and initial observations from the RECTOPET (REctal Cancer trial on PET/MRI/CT) study. Cancer Imaging 19:52. https://doi.org/10.1186/s40644-019-0237-1
2. Ghiringhelli F, Vincent J, Bengrine L, Borg C, Jouve JL, Loffroy R, Guiu B, Blanc J, Bertaut A (2019) Hepatic arterial chemotherapy with raltitrexed and oxaliplatin versus standard chemotherapy in unresectable liver metastases from colorectal cancer after conventional chemotherapy failure (HEARTO): a randomized phase-II study. J Cancer Res Clin Oncol 145:2357–2363. https://doi.org/10.1007/s00432-019-02970-8
3. Nakamura T, Ishikawa H, Sakai T, Ayabe M, Wakabayashi K, Mutoh M, Matsuura N (2019) Effect of physical fitness on colorectal tumor development in patients with familial adenomatous polyposis. Medicine 98:e17076. https://doi.org/10.1097/MD.0000000000017076

4. Park Y, Kim JS, Oh JS (2019) Zw10 is a spindle assembly checkpoint protein that regulates meiotic maturation in mouse oocytes. Histochem Cell Biol 152:207–215. https://doi.org/10.1007/s00418-019-01800-9
5. Pauleau AL, Bergner A, Kajtez J, Erhardt S (2019) The checkpoint protein Zw10 connects CAL1-dependent CENP-A centromeric loading and mitosis duration in Drosophila cells. PLoS Genet 15:e1008380. https://doi.org/10.1371/journal.pgen.1008380
6. Zhang Z, Wu L, Wang J, Li G, Feng D, Zhang B, Li L, Yang J, Ma L, Qin H (2014) Opposing effects of PI3K/Akt and Smad-dependent signaling pathways in NAG-1-induced glioblastoma cell apoptosis. PLoS ONE 9:e96283. https://doi.org/10.1371/journal.pone.0096283
7. Baek SJ, Eling TE (2006) Changes in gene expression contribute to cancer prevention by COX inhibitors. Prog Lipid Res 45(1):1–16. https://doi.org/10.1016/j.plipres.2005.10.001
8. Eling TE, Baek SJ, Shim M, Lee CH (2006) NSAID activated gene (NAG-1), a modulator of tumorigenesis. J Biochem Mol Biol 39(6):649–655. https://doi.org/10.5483/bmbrep.2006.39.6.649
9. Min KW, Liggett JL, Silva G, Wu WW, Wang R, Shen RF, Eling TE, Baek SJ (2016) NAG-1/GDF15 accumulates in the nucleus and modulates transcriptional regulation of the Smad pathway. Oncogenes 35:377–388. https://doi.org/10.1038/onc.2015.95
10. Yang MH, Kim J, Khan IA, Walker LA, Khan SI (2014) Nonsteroidal anti-inflammatory drug activated gene-1 (NAG-1) modulators from natural products as anti-cancer agents. Life Sci 100:75–84. https://doi.org/10.1016/j.lfs.2014.01.075
11. Otterpohl KL, Gould KA (2017) Evaluation of Rint1 as a modifier of intestinal tumorigenesis and cancer risk. PLoS ONE 12:e0172247. https://doi.org/10.1371/journal.pone.0172247
12. Grigaravicius P, von Deimling A, Frappart PO (2016) RINT1 functions as a multitasking protein at the crossroads betwe[illegible] genomic stability, ER homeostasis, and autophagy. Autophagy 12:1413–1415. https://doi.org/10.1080/15548627.2016.1191730
13. Passarelli MN, Barry EL, Zhang D, Gangar P, Rees J[illegible] Presalier RS, McKeown-Eyssen G, Karagas MR, Baron JA ([illegible]18) Risk of basal cell carcinoma in a randomize[illegible]linical trial of aspirin and folic acid for the prevention of colorect[illegible]adenomas. Br J Dermatol 179:337–344. https://doi.org/10.1111/[illegible]d.16571
14. Gravalos C, Carrato A, Tobena M, Rodriguez-Garrote M, Soler G, Vieitez JM, Robles L, Valla[illegible]res-Ayerbes M, Polo E, Limón ML, Safont MJ, Martínez de C[illegible] E, García-Alfonso P, Aranda E, Spanish Cooperati[illegible] Group For The Treatment Of Digestive Tumors (TTD) (2018) A [illegible]mized phase II study of axitinib as maintenance therapy after first-line treatment for metastatic colorectal [illegible]. Clin Colorectal Cancer 17:e323–e329. https://doi.org[illegible]0.10[illegible]c.2018.02.004
15. Miller DP Jr, D[illegible]izar[illegible] Thompson N, Weaver KE, Case LD, Troyer JL, Sp[illegible]gler JG, [illegible]ler D, Pignone MP (2018) Effect of a digital hea[illegible] intervention on receipt of colorectal cancer screening in vu[illegible]rable patients: a randomized controlled trial. Ann Intern Med 168:550–557. https://doi.org/10.7326/M17[illegible]15
16. Lee J, Kim I, Yoo E, Baek SJ (2019) Competitive inhibition by NAG-1/GDF-15 NLS peptide enhances its anti-cancer activity. Biochem Biophys Res Commun 519:29–34. https://doi.org/10.1016/j.bbrc.2019.08.090
17. Xiao J, Liu CC, Chen PL, Lee WH (2001) RINT-1, a novel Rad50-interacting protein, participates in radiation-induced G(2)/M checkpoint control. J Biol Chem 276(9):6105–6111. https://doi.org/10.1074/jbc.M008893200
18. Lin X, Liu CC, Gao Q, Zhang X, Wu G, Lee WH (2007) RINT-1 serves as a tumor suppressor and maintains [illegible]lgi [illegible]ynamics and centrosome integrity for cell survival. Mol Ce[illegible]iol [illegible]7:4905–4916. https://doi.org/10.1128/MCB.02396-06
19. Kadowaki M, Yoshioka H, Kamitan[illegible] Watanabe T, Wade PA, Eling TE (2012) DNA methylati[illegible]-me[illegible]ted silencing of nonsteroidal anti-inflammatory drug-activated g[illegible]e (NAG-1/GDF15) in glioma cell lines. Int J Cancer 130([illegible]):267–277. https://doi.org/10.1002/ijc.26082
20. Wang X, Baek SJ, Eling [illegible] (201[illegible]) [illegible]OX inhibitors directly alter gene expression: rol[illegible] canc[illegible]revention? Cancer Metastasis Rev 30(3–4):641–657[illegible]tps://doi.o[illegible]g/10.1007/s10555-011-9301-4
21. Yamaguchi K, [illegible]ee [illegible] Eling TE, Baek SJ (2006) A novel peroxisome proliferator-act[illegible]ted receptor gamma ligand, MCC-555, induces apopt[illegible]is via posttranscriptional regulation of NAG-1 in colorect[illegible]an[illegible]s. Mol Cancer Ther 5(5):1352–1361. https://doi.org/1[illegible]158/1535-7163.MCT-05-0528
22. S[illegible] SK, Lee J[illegible], Chon SJ, Yun BH, Cho S, Choi YS, Lee BS (20[illegible]) [illegible]ostatin A Induces NAG-1 Expression and apoptosis in human endometriotic stromal cells. Reprod Sci 25:1349–1356. https://doi.org/10.1177/1933719117741372
23. Funadani R, Sogame Y, Kojima K, Takeshita T, Yamamoto K, Tsujizono T, Suizu F, Miyata S, Yagyu KI, Suzuki T, Arikawa M, Matsuoka T (2016) Morphogenetic and molecular analyses of cyst wall components in the ciliated protozoan Colpoda cucullus Nag-1. FEMS Microbiol Lett. https://doi.org/10.1093/femsle/fnw203
24. Wen Y, Song-Ping X, Pan L, Xiao-Yan L, Shan P, Qian Y, Meng S, Xiao-Xing H, Rui-Jing X, Jie X, Qiu-Ping Z, Liang S (2019) Digital analysis of immunostaining of ZW10 interacting protein in human lung tissues. J Vis Exp. https://doi.org/10.3791/58551
25. Yuan W, Xie S, Wang M, Pan S, Huang X, Xiong M, Xiao RJ, Xiong J, Zhang QP, Shao L (2018) Bioinformatic analysis of prognostic value of ZW10 interacting protein in lung cancer. Onco Targets Ther 11:1683–1695. https://doi.org/10.2147/OTT.S149012
26. Arasaki K, Taniguchi M, Tani K, Tagaya M (2006) RINT-1 regulates the localization and entry of ZW10 to the syntaxin 18 complex. Mol Biol Cell 17:2780–2788. https://doi.org/10.1091/mbc.e05-10-0973

Publisher's Note Springer Nature remains neutral with regard to jurisdictional claims in published maps and institutional affiliations.

RETRACTED ARTICLE
